# The Use of Generative Adversarial Network and Graph Convolution Network for Neuroimaging-Based Diagnostic Classification

**DOI:** 10.3390/brainsci14050456

**Published:** 2024-04-30

**Authors:** Nguyen Huynh, Da Yan, Yueen Ma, Shengbin Wu, Cheng Long, Mirza Tanzim Sami, Abdullateef Almudaifer, Zhe Jiang, Haiquan Chen, Michael N. Dretsch, Thomas S. Denney, Rangaprakash Deshpande, Gopikrishna Deshpande

**Affiliations:** 1 Auburn University Neuroimaging Center, Department of Electrical and Computer Engineering, Auburn University, Auburn, AL 36849, USA; nph0013@auburn.edu (N.H.); dennets@auburn.edu (T.S.D.); 2 Department of Computer Sciences, Indiana University Bloomington, Bloomington, IN 47405, USA; yanda@iu.edu; 3 Department of Computer Sciences, The Chinese University of Hong Kong, Shatin, Hong Kong; yema21@cse.cuhk.edu.hk; 4 Department of Mechanical Engineering, University of California, Berkeley, CA 94720, USA; shengbin_wu@berkeley.edu; 5 School of Computer Science and Engineering, Nanyang Technological University, Singapore 639798, Singapore; c.long@ntu.edu.sg; 6 Department of Computer Sciences, University of Alabama at Birmingham, Birmingham, AL 35294, USA; mtsami@uab.edu (M.T.S.); lateef11@uab.edu (A.A.); 7 College of Computer Science and Engineering, Taibah University, Yanbu 41477, Saudi Arabia; 8 Department of Computer and Information Science and Engineering, University of Florida, Gainesville, FL 32611, USA; zhe.jiang@ufl.edu; 9 Department of Computer Sciences, California State University, Sacramento, CA 95819, USA; haiquan.chen@csus.edu; 10 Walter Reed Army Institute of Research-West, Joint Base Lewis-McChord, WA 98433, USA; dretschphd@gmail.com; 11 Department of Psychological Sciences, Auburn University, Auburn, AL 36849, USA; 12 Alabama Advanced Imaging Consortium, Birmingham, AL 36849, USA; 13 Center for Neuroscience, Auburn University, Auburn, AL 36849, USA; 14 Athinoula A. Martinos Center for Biomedical Imaging, Massachusetts General Hospital, Harvard Medical School, Charlestown, MA 02129, USA; rdeshpande3@mgh.harvard.edu; 15 Department of Psychiatry, National Institute of Mental Health and Neurosciences, Bangalore 560030, India; 16 Department of Heritage Science and Technology, Indian Institute of Technology, Hyderabad 502285, India

**Keywords:** resting-state functional magnetic resonance imaging, resting-state functional connectivity, deep learning, graph convolution network, generative adversarial network

## Abstract

Functional connectivity (FC) obtained from resting-state functional magnetic resonance imaging has been integrated with machine learning algorithms to deliver consistent and reliable brain disease classification outcomes. However, in classical learning procedures, custom-built specialized feature selection techniques are typically used to filter out uninformative features from FC patterns to generalize efficiently on the datasets. The ability of convolutional neural networks (CNN) and other deep learning models to extract informative features from data with grid structure (such as images) has led to the surge in popularity of these techniques. However, the designs of many existing CNN models still fail to exploit the relationships between entities of graph-structure data (such as networks). Therefore, graph convolution network (GCN) has been suggested as a means for uncovering the intricate structure of brain network data, which has the potential to substantially improve classification accuracy. Furthermore, overfitting in classifiers can be largely attributed to the limited number of available training samples. Recently, the generative adversarial network (GAN) has been widely used in the medical field for its generative aspect that can generate synthesis images to cope with the problems of data scarcity and patient privacy. In our previous work, GCN and GAN have been designed to investigate FC patterns to perform diagnosis tasks, and their effectiveness has been tested on the ABIDE-I dataset. In this paper, the models will be further applied to FC data derived from more public datasets (ADHD, ABIDE-II, and ADNI) and our in-house dataset (PTSD) to justify their generalization on all types of data. The results of a number of experiments show the powerful characteristic of GAN to mimic FC data to achieve high performance in disease prediction. When employing GAN for data augmentation, the diagnostic accuracy across ADHD-200, ABIDE-II, and ADNI datasets surpasses that of other machine learning models, including results achieved with BrainNetCNN. Specifically, in ADHD, the accuracy increased from 67.74% to 73.96% with GAN, in ABIDE-II from 70.36% to 77.40%, and in ADNI, reaching 52.84% and 88.56% for multiclass and binary classification, respectively. GCN also obtains decent results, with the best accuracy in ADHD datasets at 71.38% for multinomial and 75% for binary classification, respectively, and the second-best accuracy in the ABIDE-II dataset (72.28% and 75.16%, respectively). Both GAN and GCN achieved the highest accuracy for the PTSD dataset, reaching 97.76%. However, there are still some limitations that can be improved. Both methods have many opportunities for the prediction and diagnosis of diseases.

## 1. Introduction

Functional magnetic resonance imaging (fMRI) is a neuroimaging tool that measures changes in cerebral blood flow to provide a visual representation of brain activity, allowing researchers to study brain function. The use of functional connectivity (FC) obtained from resting-state fMRI (rs-fMRI) enables imaging of temporal interaction between brain regions and has therefore been extensively employed in the classification of brain disorders and the identification of objective biomarkers associated with the underlying disorders. FC is a connectivity matrix representing functional communication between different brain regions, and the strength of connection between region i and region j is represented as the value of row i and column j in the matrix. The value is calculated using Pearson’s correlation between the time series representing region *i* and *j*; however, other metrics of association between time series can also be used [1,2]. Considerable evidence from rs-fMRI studies has shown the alteration or disruption of FC in individuals with neuropsychiatric and neurodegenerative disorders [3,4,5,6,7]. Several recent works have applied convolutional neural networks (CNNs) that incorporate these altered brain FC patterns as relevant features for rapid and reliable classification of brain disorders. However, these models are constrained by two challenges. First, although traditional CNNs can extract local meaningful features from order and grid-like data (such as images), the spatial features learned in CNN may not be optimal for graph structure data (such as networks), which are invariant to node ordering and have irregular relationships between nodes. Second, patient fMRI data used for training is currently limited in its sample size because of a range of factors, such as the exorbitant expense of data acquisition, barriers to standardized data acquisition across different sites, and consequent open sharing of data. The relatively small sample size of patient data often leads to models being overfit. When relatively smaller samples of patient data are used with larger samples of healthy controls in the same model, it also causes the problem of class imbalance. To overcome those issues, graph convolutional networks (GCNs), an extended version of CNN, are proposed to deal with graph-structure data, while generative adversarial networks (GANs) can deal with data scarcity in neuroimaging due to their ability to generate additional data for training purposes.

The brain can be conceptualized as a network where the specialized regions are represented as nodes, and the pathways of communication or links between these regions are regarded as edges. By analyzing the patterns of FC, we can gain valuable insight into the temporal properties and dynamic interplay between the brain regions, revealing a more comprehensive view of the brain network. Therefore, graph theoretical analysis may be an ideal tool to investigate the organizational mechanisms underlying brain networks. Several complex graph theoretic algorithms have been applied to study the pathophysiology of various diseases [8,9,10]. The brain graph is a network representation of the intricate interactions between N distinct regions of the brain and therefore can be captured by the N × N matrix. The elements in the matrix capture the strength or degree of correlation between each pair of nodes in the network. In general, brain graphs can be categorized as functional connectivity or effective connectivity, where the former captures the strength of statistical associations or correlation between brain regions and the latter represents the directionality of information flow. Networks can also be grouped as unweighted or weighted, depending on whether the edges are assigned a binary or continuous value. In functional brain networks, the edges can be estimated by various statistical methods, such as Pearson’s correlation coefficients, Spearman’s correlation, or Kendall rank correlation coefficients.

Our research aims to design an end-to-end GCN model that can be applied to functional graphs (here, constructed from rs-fMRI data) for distinguishing healthy controls from those with brain disorders. Similar to CNN, the proposed GCN also includes a convolution operation that learns localized patterns from the networks and a pooling operation that can not only downsample the graph but also increase the receptive field, allowing the graph to learn global graph-level patterns. The model learns features from each node and its relationship with neighboring nodes to generate new feature maps via the spectral-based convolution method. The spectral convolution operation [11] can transform complex node representations to low-dimensional representations to tackle graph-structure data more easily.

To solve the problem of small sample sizes and class imbalance, we recently proposed a modified version of the existing GAN model to be able to generate realistic FC correlation matrices [12]. Generally, GAN consists of two main models that are trained in the adversarial optimization process: a generator G is designed to generate outputs that can mislead the discriminator into treating them as authentic. Unconditioned GAN or unsupervised GAN can discover the nature of data distribution and their latent structure to produce synthetic data. By utilizing those characteristics, conditional GAN and auxiliary classifier GAN have been used to allow GAN to perform classification tasks [13,14]. The classification performance can be improved by adding synthetic data to the classifier [15,16]. The proposed GAN model adapted these ideas to perform semi-supervised tasks. One of the issues involved in training GANs is the phenomenon called mode collapse, where the model only produces data belonging to a specific class. To prevent mode collapse, the proposed model utilizes supplementary information such as class category or phenotypic features to enhance the variety of the dataset. The generator of GAN will receive random noise combined with additional attributions, such as gender or age, to generate a synthetic FC matrix. The discriminator D will adopt the architecture of BrainNetCNN [17], where filters are customized to function well with the connectivity matrix. Our previous paper [12] also utilizes the inner product operation to embedding vectors to quantify the statistical link between two brain regions. Thus, we utilize the GAN we previously developed, which is an improvement over existing GAN-based methods for neuroimaging data.

We have reported on the designs of GCN and GAN needed to work on FC data and tested them on the ABIDE-I dataset [12,18]. However, there is a need to examine the generalizability of these models to other datasets derived from different patient populations. Therefore, here we will test the applicability of GCN and GAN based models on FC-based brain networks for discriminating healthy subjects from individuals diagnosed with ADHD (ADHD-200 [19] dataset), autism (ABIDE-II [20] dataset instead of ABIDE-I used in our previous work), PTSD (acquired in-house but publicly shared [21]), and Alzheimer’s (ADNI [22]) datasets. We have reported the utility of traditional machine learning models on these datasets before, and here we used those results to compare them with those obtained from GCN and GAN. We also compared the proposed models with BrainNetCNN [17] to evaluate the efficacy of GCN for extracting structural features and GAN for data augmentation. The statistical tests were also conducted to determine which models achieved superior performance.

## 2. Related Work

Deep learning has attracted considerable attention for its potential to automatically detect and classify neurological diseases at an early stage. Specifically, convolutional neural networks (CNN) have been successful in using high-dimensional medical imaging data to predict diagnostic status. Kawahara et al. [17] proposed the BrainNetCNN in 2017, which is a class of CNNs that can be used to predict non-imaging variables (such as diagnostic status) using brain networks as input features. Another study [23] improves the detection of epileptic seizures using electroencephalogram (EEG) data by applying variable-frequency complex demodulation (VFCDM) and CNNs. Building on basic CNNs, researchers have improved the classification performance by applying transfer learning, a technique that utilizes the pre-trained models to enable models to leverage knowledge gained from one dataset to perform well on different datasets [24,25,26]. This method has the advantage of allowing the model to train on image data acquired at multiple sites.

GCN is able to model the complex interconnections between nodes in a graph, making it particularly well-suited for analyzing the irregular structure of brain network data. Therefore, it has been employed for diagnostic classification using functional brain networks. Prior works proposed different GCN-based architectures to distinguish between healthy and unhealthy subjects that can be categorized as individual-based graph architecture and population-based graph architecture. The main difference between these two methods is the representation of a node, wherein nodes in the individual-based graph represent brain regions while nodes in the population-based graph denote subjects. For instance, Ktena et al. [27] proposed Siamese GCN that analyzes brain functional connectivity networks by exploiting the similarities between two brain networks with the assumption that the classification task can be significantly improved with more accurate similarity metrics. Another study used varied templates to generate brain functional/structural connectivity networks for individuals subject and then trained a triplet graph convolutional network to learn the relationship at multiple scales [28]. The proposed model achieved high performance in the classification of mild cognitive impairment and attention-deficit/hyperactivity disorder with healthy controls. On the other hand, Parisot et al. [29] considered implementing spectral GCN on a population-based graph where each subject is considered a node. The model leverages the relevant features from both rs-fMRI and non-imaging data to discriminate between nodes of healthy control and nodes of individuals with autism disorder. Kim et al. [30] introduced the spatio-temporal attention graph isomorphism network (STAGIN) model, which addresses dynamic graphs by employing two spatial attention READOUT mechanisms (Graph-Attention READOUT (GARO) and Squeeze-Excitation READOUT (SERO)) to capture spatial features at each time point and employing a transformer encoder to learn temporal attended features. Zhao et al. [31] introduced a data augmentation approach combining a “sliding window” strategy with the self-attention mechanism GCN (SA-GCN) for autism classification, utilizing time series subsegments to construct correlation matrices, and introducing both low-order and high-order functional graphs to enable the model to exploit features from various perspectives. Another study [32] proposed a model that comprises two distinct GCNs, f-GCN and p-GCN, where f-GCN analyzes individual brain networks within subjects by utilizing stacked GCNs and eigenpooling for coarsened graph generation, employing max pooling for node representation aggregation, while p-GCN, a population-based model, treats each subject as a graph node and utilizes f-GCN output as a node feature.

Researchers have applied the generative aspect of GAN to various tasks in medical image analysis, including classification [33], segmentation [34], de-noising [35], image reconstruction [36], and image synthesis [37]. The use of GAN as a data augmentation method has been shown to outperform various traditional augmentation methods. GAN with feature matching has been proposed to discriminate psychiatric patients from controls [38]. The model learns to generate functional network connectivity that is constructed by independent component analysis, and the feature matching technique was used to stabilize the training process. The paper shows that GAN performs better than other traditional machine learning methods, such as support vector machine or nearest neighbors, with more than 6% higher accuracy. Barile et al. [39] utilized GAN with an autoencoder to generate brain connectivity for multiple sclerosis (MS) classification, ensuring that the model’s training prevents collapse by producing synthetic data matching real data statistics. Cao et al. [40] introduced a multiloop algorithm aimed at improving the quality of generated data by enabling the assessment and ranking of sample distribution in each iteration, facilitating the selection of high-quality samples for training. While many studies have focused on generating realistic 3D brain images, only a few studies have developed GAN models to learn to mimic functional connectivity networks. This is not only computationally less demanding but also helpful in understanding brain network anomalies and underlying brain disorders.

## 3. Material and Methods

### 3.1. Data

**Attention deficit hyperactivity disorder (ADHD)** ADHD is a prevalent neurobehavioral disorder in childhood that is typically characterized by symptoms of inattention, hyperactivity, and impulsivity. Children with ADHD are classified into three separate categories: ADHD-I (inattention), ADHD-H (hyperactive/impulsive), and ADHD-C (combination of both symptoms). The ADHD-200 Global Competition was held in summer 2011 and challenged teams to provide the best performance for diagnosing individuals with ADHD from their resting-state fMRI scans [19]. There are 929 subjects in the dataset, which consists of 573 healthy controls, 207 individuals with ADHD-C, 13 individuals with ADHD-H, and 136 individuals with ADHD-I. Scanning for each participant took place at one of seven distinct sites, namely Peking University, Kennedy Krieger Institute, NeuroIMAGE Sample, New York University Child Study Center, Oregon Health & Science University, University of Pittsburgh, and Washington University. For more information regarding the acquisition parameters and site distribution, please refer the webpage http://fcon_1000.projects.nitrc.org/indi/adhd200/, accessed on 19 March 2024. Since there are fewer subjects diagnosed with subtype ADHD-H in comparison with the other classes, we combined subjects with ADHD-H into ADHD-C, which makes the problem into a 3-way diagnosis classification.

**Autism Spectrum Disorder (ASD)** ASD is a clinical term that encompasses a range of neurodevelopmental disorders marked by deficits in social behavior and communication skills, along with repeated behaviors and restricted interests. The classification of ASD individuals was carried out using an rs-fMRI image from the Austim Brain Imaging Data Exchange Data (ABIDE). ABIDE is a group of organizations that has collected and distributed datasets containing rs-fMRI, alongside additional clinical and demographic information from both individuals with ASD and those who are typically developing [20,41]. The initial ABIDE data, or ABIDE I, have been experimented with by the two models in the papers. In this work, the algorithms were extended to apply to ABIDE II, a new multi-site open data resource that was established to increase the sample size. Data for the imaging were obtained from 11 different facilities and involved a total of 623 participants. Of these, 356 were considered to be healthy conhorts, 214 had been diagnosed with autism patients, and 53 had been diagnosed with Asperger’s syndrome (a mild symptom of autism).

**Post-traumatic stress disorder (PTSD) & post-concussive syndrome (PCS)** PTSD is a psychological disorder that develops in some individuals who have experienced shocking, horrifying, or life-threatening events. PCS is a condition in which symptoms or other functional difficulties persist for a period of time after sustaining a concussion or a mild traumatic brain injury. Such disorders often co-occur in individuals serving in the military. This study investigating PTSD/PCS involved 87 active-duty US solders recruited from Fort Moore, GA and Fort Novosel, AL, USA. Data collection was approved by the Institutional Review Board (IRB) at Auburn University and the U.S. Army Medical Research and Development Command IRB (HQ USAMRDC IRB). This sample included 28 combat controls, 17 individuals diagnosed with PTSD, and 42 individuals who had both PTSD/PCS. The imaging data for the study were obtained exclusively at the Auburn University Neuroimaging Center. Information about screening procedures to diagnose PTSD/PCS symptoms and acquisition parameters can be found in the paper [21]. Since each subject has 2 runs, we will treat each run as 1 subject, resulting in a dataset with 174 subjects in total.

**Mild cognitive impairment (MCI) & Alzheimer’s disease (AD)** As people age, the risk of developing AD increases, and this condition is the primary cause of dementia in the US. When an individual experiences mild cognitive dysfunction in the memory domain, they may be diagnosed with MCI, and it is believed that people who are diagnosed with MCI are at an increased risk of developing AD later in life. Diagnosis and treatment of the condition remain challenging, with no definitive diagnostic test and cure available at present. Therefore, accurate detection of MCI can aid in preventing further deterioration and slowing the progression of AD. The imaging data was sampled from the Alzheimer’s disease neuroimaging initiative (ADNI) database to perform a 4-way multiclass classification: healthy controls, early MCI (EMCI), late MCI (LMCI), and AD. In particular, 35 matched healthy controls, 34 subjects with EMCI, 34 subjects with LMCI, and 29 subjects with AD were collected from the database. The data acquisition process used for this study can be found in the paper [22].

### 3.2. Data Preprocessing

FC was derived with the assistance of Data Processing Assistant for Resting-state MRI (DPARSF, version V5.3_210101) and functional connectivity toolboxes (CONN) softwares, version v.22.a (https://web.conn-toolbox.org/, accessed on 19 March 2024). Firstly, to minimize subject motion artifacts during the scanning process, motion correction techniques were performed to align each image to a standard reference point in time. Then, slice time correction was performed, and after that, the subject’s data underwent a nonlinear transformation to align it with a common reference MNI152 (Montreal Neurological Institute) space, which facilitates group-level analysis. The preprocessing pipeline also includes regressing out nuisance variables, such as six head motion parameters, the mean white matter, and the cerebrospinal fluid (CSF) signal, in order to minimize confounding effects. Then, the estimation of the underlying neural time series was carried out using the blind deconvolution method proposed by Wu et al. [42]. The deconvolved data was then achieved by the Wiener filter. We applied a temporal band-pass filter with a bandwidth of 0.01–0.1 Hz to the data. Mean time series was extracted from defined 200 regions of interest provided by Craddock (known as the CC200 template) [43]. Pearson’s correlations between the mean time series of two brain regions were established, resulting in the FC for each subject with shape 200 × 200. However, due to incomplete brain coverage in the ADHD data, only 190 out of 200 regions were captured using the Craddock atlas. Similar to the ADHD dataset, the PTSD dataset suffered from incomplete data coverage and was only able to cover 125 out of 200 regions.

### 3.3. Graph Convolutional Network

**Figure 1:**
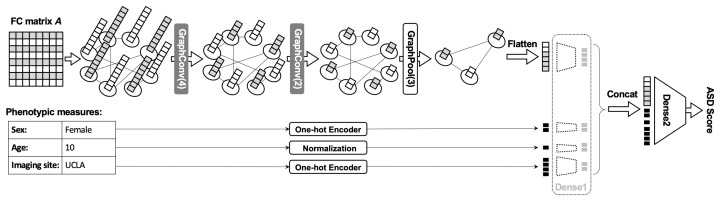
Illustration of the GCN architecture proposed in our previous work [18] that we have applied here. In the figure, the model consists of two convolutional layers that transforms the number of node features from 8 to 2 and one pooling layer that pools the number of nodes from 8 to 3. The output of GCN was also concatenated with subject’s attribute data (gender, age, imaging site) and then the combined input was passed to the classifier. The results reported in this paper were generated by this GCN architecture with a slight changes in parameters in each layer (as described in methods).

The GCN architecture is depicted in Figure 1. For each subject, we define an undirected graph G≡{V,E} as a functional brain network, where $V=\{v_{1},\cdots v_{i}\}$ is a set of N nodes (N may vary depending on the number of regions of interests) and $E=\{e_{ij}\}$ represents a collection of connectivity edges from node $v_{i}$ to node $v_{j}$. The graph was represented by an adjacency matrix **A**$\in R^{N\times N}$, where each element $a_{ij}=1$ if the value of the corresponding position of the mean matrix **$\bar{A}$** is greater than the cutoff threshold τ and $a_{ij}=0$ otherwise. The mean matrix **$\bar{A}$** was determined by the mean of all the functional connectivity matrices in the training dataset, and the threshold τ was decided by the percentage of positive connections that we need to keep. One of the reasons that support this idea is that by taking the mean, we can sparsify the data to different degrees by varying the threshold. Furthermore, by keeping only relevant connections between regions, we can detect abnormal changes in meaningful patterns or connections that can effectively separate healthy subjects and subjects with brain disorders [3,4,5,6,7].

In this work, the graph convolutional layer was implemented from the spectral perspective. In the process of spectral graph convolution, the graph signals are transformed from node domain to frequency domain using the graph Fourier transform. Then, to reduce the computational complexity and enable the graph to learn locally, the K-polynomial filters were used in ChebNet; this approach can be simplified by taking only the first order approximation [11]. Hence at layer l, the output representation node was computed as:

(1) $$H^{\left(l\right)}=\sigma \left({\tilde{D}}^{-\frac{1}{2}}\tilde{A}{\tilde{D}}^{-\frac{1}{2}}H^{\left(l-1\right)}W^{\left(l\right)}\right)$$

where $\tilde{A}=I+A$ is equivalent to adding self-loops to the adjacency matrix and $\tilde{D}$ is the diagonal degree matrix of $\tilde{A}$, i.e., ${\tilde{D}}_{i,i}=\sum_{j}{\tilde{A}}_{ij}$. σ is activation function (Rectified Linear Unit (ReLU) or linear activation function). In this work, ReLU activation was chosen. Furthermore, $H^{\left(l-1\right)}\in R^{N\times d}$ represents d attributes of the N nodes, and $W\in R^{d\times m}$ refers to a learnable matrix used at layer *l* that transforms the input node representation $H^{\left(l-1\right)}$ from d to m feature dimensions. The initial node representations $H^{\left(0\right)}$ are just the original input features or functional connectivity of each subject: $H^{\left(0\right)}=X$. As evident, we employed an individual-based graph architecture. Equation (1) aggregates node representations in their direct neighborhood, helping to gain more information after each iteration for the purpose of learning the graph.

To apply GCN to the graph classification task, a graph-level representation is needed. Similar to conventional CNNs where pooling method is applied to reduce the spatial resolution, many methods of pooling for GCNs have been proposed with the aim of decreasing the number of nodes to obtain coarser graphs while preserving important graph properties. One of the graph pooling approaches is self-attention graph pooling (SAGPool), which is a technique that utilizes a graph neural network to produce a score for each node based on its features, and subsequently selects the K nodes with the highest score [44]. Specially, the self-attention scores z for each node is calculated as:

(2) $$z=\tanh({\tilde{D}}^{-\frac{1}{2}}\tilde{A}{\tilde{D}}^{-\frac{1}{2}}H^{\left(l-1\right)}\Theta^{\left(l\right)})$$

where $\tilde{A}=A^{\left(l-1\right)}+I$, which depends on the adjacency matrix of the previous layer, and $\Theta \in R^{d\times 1}$ is the weight of the pooling layer. Because graph pooling changes the graph or particularly the adjacency matrix A, the shape of adjacency matrix A and the output node representation after pooling will change based on the top-k nodes we want to keep. To update those variables, first the top-k nodes were obtained as the following steps:

(3) $$\begin{array}{cc} \mathrm{idx} & =\mathrm{top}\text{-}\mathrm{rank}(z,k) \end{array}$$

(4) $$\begin{array}{cc} z_{mask} & =z(\mathrm{idx}) \end{array}$$

The outputs of graph pooling were then determined as:

(5) $$\begin{array}{cc} H^{\left(l\right)} & =H^{\left(l-1\right)}\left(\mathrm{idx},:\right)⊙z_{mask} \end{array}$$

(6) $$\begin{array}{cc} A^{\left(l\right)} & =A^{\left(l-1\right)}\left(\mathrm{idx},\mathrm{idx}\right) \end{array}$$

where $H^{\left(l-1\right)}\left(\mathrm{idx},:\right)$ contains node-specific features that are indexed, ⊙ performs element-wise multiplication, and $A^{\left(l-1\right)}\left(\mathrm{idx},\mathrm{idx}\right)$ is an adjacency matrix that is indexed by both rows and columns.

Non-imaging measures that contribute variance to the imaging data, such as gender, age, and imaging site, can also combine with the extracted features from GNN to boost the prediction performance. To guarantee that all feature values are bounded in the interval [0, 1], gender and imaging site features were first encoded to one-hot vectors, while the age feature was normalized by dividing by 100. All non-imaging features were also transformed to the vector of length 2 by the dense layer, and 1 dense layer was also used to transform the output of the GNN model to the vector of length 15. Those vectors were then concatenated and used as input for the classifier that consists of one dense layer with a softmax activation function to compute the likelihood of each subject’s network belonging to a particular class label.

### 3.4. Generative Adversarial Network

**Figure 2:**
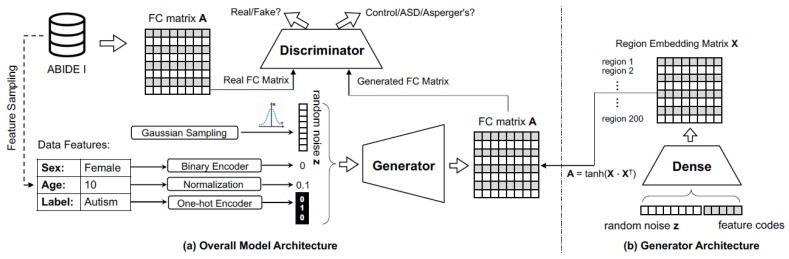
Illustration of the GAN model proposed by using previously [12], which we have used in this work. The generator produces a synthetic functional connectivity matrix via the combined input of random noise and feature codes (gender, age, and label). The discriminator was trained on both real FC data and synthesized FC data generated from the generator.

Generative adversarial network (GAN) comprises two different functional models, namely the discriminator (D) and the generator (G). The two models can be trained simultaneously, in which the generator takes random variable z from a prior distribution (usually Gaussian noise or uniform distribution) to generate new images, while the discriminator focuses on distinguishing whether the image is authentic or not. For supervised learning, the output of the discriminator will also include the probabilities of the class label in addition to its validity output. GAN is able to generate synthetic data that are of high quality and closely resemble real data by using an iterative adversarial approach. The specific designs of the discriminator and the generator are demonstrated in the following (and visually illustrated in Figure 2):

**Generator architecture**: The generator collects the random noise vector z drawn from a uniform distribution to produce synthetic functional connectivity data. One of the issues of the generator is mode collapse, which occurs when there is only a limited set of samples that the generator can generate. To mitigate this problem, we use ideas from conditional GAN (CGAN) [13] and InfoGAN [45], which integrate more attribute data into the latent input, including category labels and phenotypic measures (such as age, gender, etc.).

Typically, the generator will directly output the image from the latent input, which will violate the nature of functional connectivity, where each entry in the matrix corresponds to the correlation coefficients between the average time series of pairs of brain regions *i* and *j*. By transforming the latent vector z to a X matrix where $X\in R^{N\times d}$, we will have each row in X representing the embedding vector of one brain region (N is the number of ROIs and d is the dimension of the embedded region). Then the generated output A is determined by taking the inner product of X with tanh activation function to ensure each value in A will have a range from −1 and 1:

(7) $$A=\tanh(XX^{T})$$

**Discriminator architecture**: The discriminator is provided with both types of inputs—the original image or a synthesized one—and decides whether the input is real or not. To boost the performance of the discriminator, phenotypic features for each subject were also included as input besides the FC matrix. Similar to the design of deep convolutional GAN (DCGAN) [45], which uses multiple convolution layers to extract features, we employed BrainNetCNN, which was proposed as specifically designed convolutional filters for modeling brain networks. The BrainNetCNN consists of three special convolution layers: the edge-to-edge layer (ECE), the edge-to-node layer (ECN), and the node-to-graph layer (NCG). The ECE layer used cross-shaped filters to calculate the weighted sum of all the neighboring edges that results in a new edge value. On the other hand, regarding edge-to-node layer, given one node, we do the convolution for all the edges that connect to that node. If the number of ROIs is N, then the output of the ECE layer will have the shape of N×N, while the shape of the output of the ECN layer is N×1. Finally, the NCG layer acts as a fully connected layer, which summarizes all the nodes into a single graph.

Then the dense layers were used to convert the output of the NCG layer and phenotypic features to a new feature space. These two vectors were then concatenated and fed to the dense layer with two heads, one with sigmoid activation for validity classification and another with softmax activation for label classification.

## 4. Experimental Setting

The architectures and hyper-parameters of both GAN and GCN were adopted from our previous papers [12,18] based on their highest performances on the ABIDE-I dataset.

In particular, the GCN model that was tested on the datasets has the following structure: 2 convolution layers, followed by 1 pooling layer. In particular, the first and second convolution layers transformed feature vectors to have sizes of 25 and 10, respectively, then the pooling layer was applied to downsample the graph from N nodes to 10 nodes. The shallow GCN was selected because the model performance tends to decrease with an increase in the number of layers. This phenomenon is known as over-smoothing, where through many messages passing steps, all node representations may become similar to each other, making it infeasible to identify discriminant features. The output of the pooling layer is then flattened and integrated with normalized age, one-hot coding of gender, and the imaging site (only available for ADHD and ABIDE-II datasets). One classifier layer was used to directly read out the combined inputs to produce the probability for each class by using the softmax activation function.

Regarding GAN, the discriminator has three type of layers similar to BrainNetCNN, which include an ECE layer with 16 feature maps, followed by an ECN layer with 64 filters, and an NCG layer with 128 filters to extract all the nodes’ features. The BatchNormalization, the LeakyReLU activation function, and the Dropout function with a dropout rate of 0.5 were used consecutively after each layer. The dense layer with 64 hidden units continues to extract features from the flattened output of the NCG layer. To combine with phenotypic features, the age and gender of one individual are first concatenated to a vector of length 2, and this vector is then transformed into a vector of length 16 by a dense layer. The fully-connected output is then merged with this feature vector. The combined input is passed through one more dense layer with 32 perceptrons before being fed to the classification layer that predicts the class label for the subject as well as the validity of the FC (real or fake). As for the generator part, a random vector of length 50 (including gender, age, and label) is fed into the embedding layer, which has the function to turn the input into an N×d matrix, where N corresponds to the number of regions and d represents the embedded dimension. N are equal to 190, 200, 125, and 200 for the ADHD, ABIDE-II, PTSD, and ADNI datasets, respectively, while d is selected to be 10. Since not all subjects in the ADHD and PTSD datasets had usable data from all 200 ROIs (either because of data quality or a lack of whole-brain coverage), the values of N for these datasets are not equal to 200. Nonetheless, the left-out ROIs corresponded to the cerebellum, and subcortex and cortical ROIs were present in all datasets. For every region, its feature representation is stored in a single row of the matrix. The inner product is then taken to output the functional connectivity matrix.

A test dataset consisting of 10% of the data was created for each dataset to assess the model’s performance. After leaving out 10% of the data for testing, a 5-fold cross-validation approach was used to split the remaining data into training and validation sets. Therefore, each model was trained five times, and the cross-validation performance of each model is the average of these repeated runs. The model that had the best performance on the validation set was chosen for assessment on the test set. The test accuracy is, of course, obtained by using the test data on the trained model once. For the GAN model, validity accuracy is also considered to select the model besides its performance on the validation set (note that in GANs, the discriminator has two outputs: one for the probability of validity to test the authenticity of the FC (real or fake) and one for classification (HC or patients)). We applied the Adam algorithm as an optimization method with a learning rate of 0.01 for GCN and a learning rate of 0.0001 and $\beta_{1}=0.5$ for GAN.

**Other models**: For comparison purposes, 18 traditional machine learning models used by Lanka et al. [21] were also trained on all the datasets by the default hyper-parameters from Scikit-learn and Matlab tools provided in the paper. These models include probabilistic or Bayesian methods. In the probabilistic framework, the models were assumed with some prior belief in the data distribution, and then the model parameters were selected to maximize the probability of the observed data, given particular parameter settings. The representatives of the probabilistic models were Gaussian Naïve Bayes (GNB), linear discriminant analysis (LDA), quadratic discriminant analysis (QDA), sparse logistic regression (SLR), and ridge logistic regression (RLR). The kernel-based models utilize kernel functions to transfer the input into a different space, and then the models can be trained on the new feature space, including support vector machines with linear functions (LinearSVM), radial basis functions (RBF-SVM), and relevance vector machines (RVM). Some traditional neural networks are also involved, namely the multilayer perceptron neural net (MLP-Net), the fully-connected neural net (FC-Net), the extreme learning machine (ELM), and the linear vector quantization net (LVQNET). Also, k-nearest neighbors (kNN) is an instance-based learning model that assigns the unknown data to the appropriate categories based on the distances between the unknown data and the data points that have been labeled. Finally, ensemble learning is the technique that allows multiple classifiers to solve a problem with the belief that multiple classifiers can provide a better result than a single classifier. Using a decision tree as a base classifier, several methods were used to train ensemble classifiers, namely bagged trees, boosted stumps, random forest, and rotation forest. Further details regarding these models can be found in Lanka et al. [21]. Additionally, BrainNetCNN, which is the top-performing method for connectome classification, was also trained with the same 5-fold CV, and the hyper-parameters and training process are similar to the settings of the discriminator in GAN.

To evaluate the models, using only accuracy may not be appropriate for imbalanced classification scenarios. Therefore, other metrics such as precision score, recall score/sensitivity, specificity, F1 score, and area under the curve (AUC) are also reported. Those metrics often apply to binary classification problem; therefore, to deal with multiclass classification, the one-vs-rest (OvR) algorithm (with a macro-averaging strategy) was used.

## 5. Results

### 5.1. Cutoff Threshold

**Figure 3:**
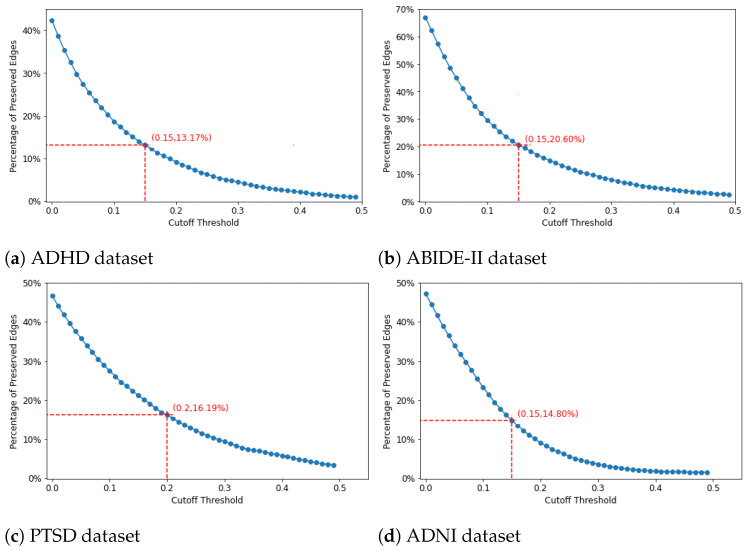
Percentages of edges preserved when the cutoff threshold is varied for each dataset.

The binary adjacency matrix representing the graph for each dataset was built by thresholding the values of the mean matrix derived from the training data. In particular, if the correlation coefficient between region *i* and region *j* is greater than cutoff threshold τ, the value of the adjacency matrix at (*i*, *j*) is equal to 1 and 0 otherwise. In order to choose the appropriate threshold, we plotted the percentages of preserved edges against the cutoff threshold and chose the elbow of the curve as the cutoff, as in previous work [46,47]. The mean matrix was derived from the average of all the training data across the 5-fold CV. Figure 3a–d shows the appropriate cutoff thresholds that can preserve meaningful edges for the ADHD, ABIDE-II, PTSD, and ADNI datasets, respectively. The cutoff threshold for ADHD, ABIDE-II, and ADNI datasets is 0.15, which maintains 13.17%, 20.60% and 14,80% of the total edges in each dataset, respectively, while the threshold for the PTSD dataset is 0.2, which keeps 16.19% of edges.

### 5.2. Model Comparison

**Figure 4:**
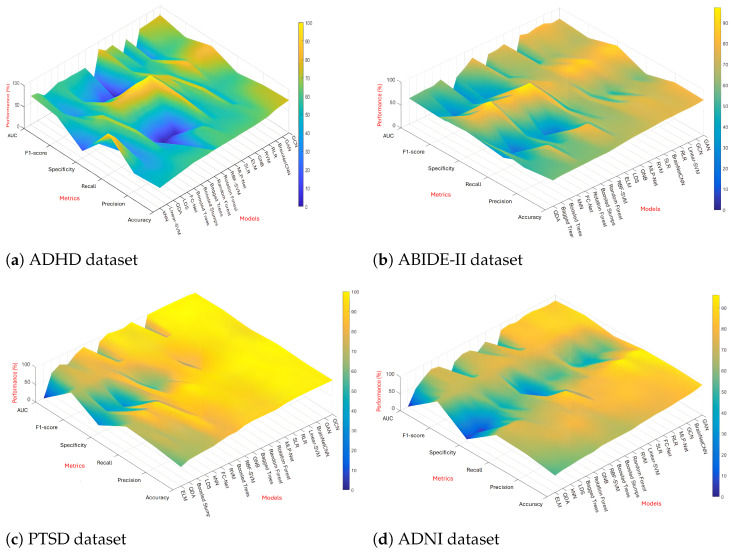
Illustration of the models’ performance sorted from worst to best for each dataset.

The outcomes of all the models for multinomical classification are presented in Table 1 (a), Table 2 (a), Table 3 (a), and Table 4 (a) for the ADHD, ABIDE-II, PTSD, and ADNI datasets, respectively, while Table 1 (b), Table 2 (b), Table 3 (b), and Table 4 (b) demonstrate the results of those respective datasets in binary classification scenario. The value highlighted with red color represents the top performing result across all the models, while the blue highlight indicates the second highest result. In Figure 4, the models have been sorted from worst to best performance. We can observe that some models may perform very well for some metrics or datasets, but the deep learning models (including GCN and GAN) generally perform well across all metrics and datasets.

**ADHD** For multinominal classification, GCN achieves the highest values for the accuracy score, precision score, and f1 score and the second highest for AUC. GAN also achieves the second highest accuracy score with 68.16%, which is only 3% less than the accuracy of GCN. The results remain the same in the binary classification scenario, with the only exception in the precision score where the GAN model takes the first place while GCN has the second place. Although the RBF-SVM model has the highest performance for specificity and AUC scores, its recall score is rather low with only 1.67%, which fails to predict the actual patients with disease. GAN and GCN therefore achieve better performance overall among all the models.

**ABIDE-II** GAN and GCN outperform the other models in accuracy for both multinomial classification (73.56% and 72.28%) and binary classification (77.40% and 75.16%). GAN also shows the highest results in precision score and f1 score. kNN, RBF-SVM, and random rorest classifiers obtained the highest and second highest specificity; however, their recall scores are rather low. On the other hand, the specificity scores of GAN and GCN are relatively high (88.34% and 88.9% respectively).

**PTSD** This is a homogeneous dataset wherein the scanning of all subjects was carried out on a single scanner using the same sequence. Since the sources of non-neural variability are minimized relatively in this dataset, most models performed very well (AUC > 90%). Therefore, it is not very informative to evaluate various classification models against one another. Nevertheless, BrainNetCNN outperforms GAN and GCN in terms of accuracy, precision, and f1 score for 3-way classification. Also in 3-way classification, while the evaluation results of GCN were outperformed by Linear SVM and BrainNetCNN, the model still has better performance than the others do (by a margin of 1% to 4%). As for binary classification, it can be seen that GAN and GCN have approximately similar patterns where they achieve the highest accuracy, highest recall, highest f1 score (97.76%, 100% and 98.40% respectively), and second highest precision score (96.92%) and specificity (93.33%). The best performance on this dataset also includes RLR, Linear SVM, and BrainNetCNN.

**ADNI** GAN appeared to reach the top level of performance in both 4-way classification and binary classification, particularly the accuracy score where the value is higher than the second highest value by large margins (52.84% vs. 44.28% and 88.56% vs. 82.86%). GCN displays only the second highest result in accuracy for multinomial classification. The reasons for this issue may be due to the limited sample dataset for training and the fact that the cut-off threshold may remove some important features in the graph.

### 5.3. Effect of Different Thresholds on GCN’s Performance

**Figure 5:**
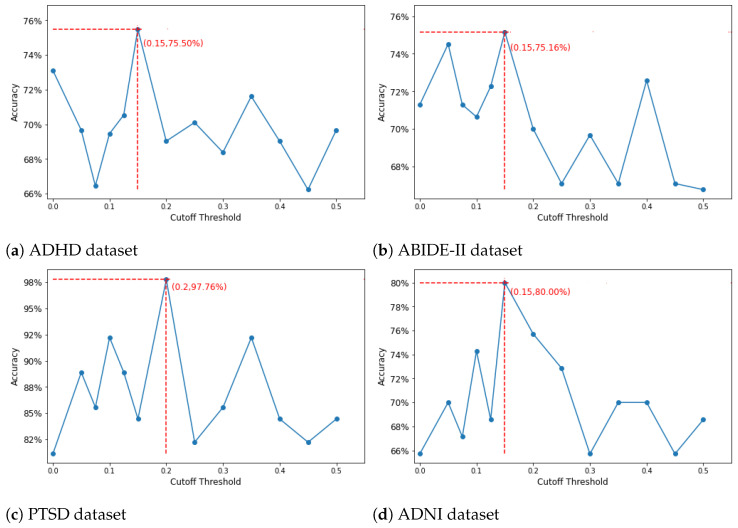
GCN’s performance on different thresholds for each dataset.

Even though we have used a criterion for threshold selection that has been widely reported before, we want to ensure that our choices do not remove any important connections that may negatively impact the model’s performance. Therefore, we estimated binary classification for the four datasets and plotted against different cutoff thresholds. As we can see in Figure 5a–d, all the accuracy results for all four datasets peak at our choices of thresholds, justifying the selection of thresholds based on the elbow cutoff criterion.

### 5.4. Statistical Significance

A random classifier for the binary classification problem would have the probability of 50% to predict the label correctly. A model with a prediction below that expectation cannot be used [48]. Therefore, we modeled the outcomes of each classifier as a Bernoulli process B(n,p), where n is a total number of subjects from the test samples and p is the probability of success. Then we want to test whether the probability of correctly predicted labels by the classifiers could surpass the expected probability. The results of all the models on all the datasets are shown in Table 5. GAN and GCN appear to achieve significant results on all the datasets.

### 5.5. Statistical Comparison

To test the hypothesis that GAN and GCN generalize better than the other models, all the accuracy scores generated by the CV method were collected as samples for a statistical test. In particular, we made the assumption of the null hypothesis that the performances of GAN and GCN are worse than those of the other models, and we would like to check whether there is enough evidence to reject the null hypothesis. The Wilcoxon rank-sum test was applied to compare the performances of GAN and GCN with other models. The Wilcoxon technique, as an alternative approach to the Student’s *t*-test, can be more appropriate when the sample is small because we cannot assume the data are normally distributed [49]. The level of significance was selected at α=0.05.

Table 6 (a) and (b) show the statistical results (*p*-value) of the Wilcoxon test for the comparison of GAN and GCN, respectively, with the other models on all the datasets. The tests indicated that GAN and GCN statistically have greater accuracy scores than almost all the traditional ML models on all the datasets (*p*-value < 0.05). We also do not have enough evidence to conclude that GAN and GCN statistically perform better than BrainNetCNN, although the test suggests that GAN has a better performance than BrainNetCNN for the ABIDE-II dataset (*p*-value = 0.02).

## 6. Discussion

GAN shows excellent results on independent test data on both large and small datasets, where the model had the best performance for the ABIDE-II, PTSD, and ADNI datasets and the second best performance for the ADHD dataset. The improvement of GAN using BrainNetCNN as the backbone network over using just BrainNetCNN alone demonstrates the benefits of data augmentation by GAN. This could potentially address the problem of data scarcity for neuroimaging based diagnostic prediction in patient populations in neurology and psychiatry.

Table 7 shows the computational time required for each model to complete training across datasets. Generally, all three deep learning models require more time to train than the traditional method, which can be attributed to their complexity and larger number of trainable parameters. We can observe that the GAN exhibits the longest training time. This is because the GAN model needs to learn the data distribution to synthesize data, in addition to the time required for training the classifier. Despite this extended training time, GAN achieves the best performance among all models across the four datasets. Notably, GCN requires less training time than BrainNetCNN across the three datasets (ABIDE-II, PTSD, and ADNI), yet it achieves better performance in ABIDE-II and ADNI and equivalent performance in PTSD. This suggests that, despite requiring fewer trainable parameters, GCN is a superior tool for capturing the complex structure of brain networks. Some traditional models require very little training time, sometimes as low as 0.01 s. However, their performance does not match that of GAN and GCN. This indicates a trade-off between training time and performance across traditional and deep learning models. In future research, there is a need to decrease the training time of GAN and GCN while maintaining satisfactory accuracy results to enhance their practical applicability in real-world clinical settings.

In Figure 3a–d, we can see that each dataset has a different cut-off threshold. As mentioned above, we aim to retain only the strong connections in the backbone network crucial for identifying abnormal patterns in individuals with brain disorders. Therefore, we intend to prune the low tail of the curve, which comprises solely low connection values. However, selecting an excessively high threshold may result in the elimination of many relevant connections, thereby negatively impacting accuracy performance (as demonstrated by examples in Figure 5a–d, where accuracy decreases with increasing thresholds). To strike a balance, we opt to set the threshold at the elbow of each curve distribution, which shares a similar concept with the elbow criterion used in k-means clustering. This choice allows for the retention of meaningful connections while removing redundant, noisy ones. Our hypothesis is validated by the accuracy results presented in Figure 5. Additionally, since each dataset exhibits distinct distributions in connection values, the selection of the elbow must vary accordingly. This accounts for differences in cut-off threshold selection across datasets.

## 7. Limitations and Future Research

The hyperparameters used in this paper were obtained from our previous works [12,18], where a hyperparameter tuning approach was employed to select the optimal parameters yielding the best results. Therefore, we applied the same parameters to this paper and achieved good results. However, it must be noted that extensive tuning of hyperparameters to a given dataset makes the model overfit the data and hence makes it less generalizable. This is not desirable in clinical diagnostic applications since there is wide variability in the human population, and we want these models to be generally applicable.

Ensemble methods can combine multiple deep neural networks to achieve more stable and generalizable predictions by mitigating variance and reducing generalization errors. However, due to the distinct characteristics and nature of GANs and GCNs, the development of ensemble frameworks for these techniques remains incomplete. While implementing this method requires careful planning and a significant time investment, its potential benefits are substantial. In our future work, we aim to explore the integration of GANs and GCNs to investigate whether this combination can lead to further performance improvements in terms of accuracy.

Interpretability is considered a crucial factor when integrating deep learning into clinical practice. In our study, we employed GCN coupled with a top-k pooling method. This approach offers interpretability by selecting a set (k) of the most relevant brain regions most predictive of brain disorders. These identified regions have the potential to serve as biomarkers, helping in the early detection of diseases. Although the paper has not presented the results, the methods hold significant potential, and we plan to implement them in future work.

GCN illustrates the effectiveness of applying graph neural networks to graph-structure data by achieving the highest performance in the ADHD dataset and also comparatively good results in other datasets. One of the ways to improve GCN is to train embedding of nodes in a space that has fewer dimensions instead of directly using row vectors as feature vectors [50]. This technique utilizes a framework from an encoder-decoder perspective that can better capture the information contained in the data. The design of the adjacency matrix also plays an essential role. Instead of static non-directional graphs obtained from FC, directional graphs can be obtained using effective connectivity [51]. The graphs could also be computed across different blocks of time to estimate the dynamics [52]. These types of advanced graphical features, when used with GCN, have the potential to improve our understanding of the mechanisms underlying neuronal dynamics by examining alterations between patients and healthy controls.

## 8. Conclusions

We identified two major challenges for the application of deep learning for neuroimaging-based diagnostic classification: small sample sizes of patients and incompatibility of graphical features of brain networks and architectures of traditional deep learning models. We have illustrated how these issues can be addressed using brain connectivity features from four different clinical datasets. The patient data scarcity issue was addressed using GANs, while GCNs allowed us to conveniently handle graph-based features within a deep learning framework. Both GAN and GCN provided the best and second best accuracy for the four clinical datasets we used.

## Figures and Tables

**Table 1:** Performance comparison of models on ADHD dataset for multinomial (**a**) and binary (**b**) classification (Red color indicates best performance, while blue color denotes second best performance).

			(**a**)			
**Model**	**Accuracy**	**Precision**	**Recall**	**Specificity**	**F1 Score**	**AUC**
GNB	54.19%	33.72%	47.86%	56.49%	39.56%	68.48%
LDS	50.32%	19.55%	13.51%	72.28%	15.47%	55.81%
QDA	44.52%	17.47%	18.31%	63.51%	13.15%	49.89%
SLR	59.35%	24.93%	22.08%	80.00%	23.18%	75.42%
RLR	62.15%	35.18%	37.99%	73.68%	36.34%	75.31%
Linear SVM	41.72%	33.67%	51.49%	31.93%	40.58%	68.13%
RBF_SVM	61.94%	30.00%	1.36%	100.00%	4.35%	82.28%
RVM	63.44%	42.24%	22.86%	86.67%	29.31%	-
MLP-Net	52.47%	32.62%	50.19%	50.88%	39.34%	71.49%
FC-Net	45.38%	22.96%	33.34%	49.47%	26.09%	61.38%
ELM	57.63%	34.43%	32.21%	71.58%	33.24%	-
KNN	33.76%	13.41%	50.00%	16.49%	21.16%	71.25%
Bagged Trees	57.42%	14.52%	3.44%	91.23%	6.91%	57.46%
Boosted Trees	57.42%	20.41%	15.45%	81.75%	17.49%	57.81%
Boosted Stumps	57.63%	28.97%	14.74%	83.86%	19.36%	63.52%
Random Forest	61.29%	0.00%	0.00%	100.00%	-	59.80%
Rotation Forest	61.29%	22.67%	2.73%	97.89%	7.82%	-
BrainNetCNN	67.74%	53.32%	42.60%	82.44%	46.08%	74.96%
GAN	68.16%	41.16%	34.82%	85.96%	36.82%	74.46%
GCN	71.38%	59.52%	45.00%	84.58%	49.86%	75.94%
			(**b**)			
**Model**	**Accuracy**	**Precision**	**Recall**	**Specificity**	**F1 Score**	**AUC**
GNB	63.23%	51.78%	73.89%	56.49%	60.88%	69.38%
LDS	54.84%	38.17%	27.22%	72.28%	31.76%	54.11%
QDA	52.47%	37.79%	35.00%	63.51%	36.16%	49.25%
SLR	62.80%	52.97%	35.56%	80.00%	42.53%	73.68%
RLR	66.02%	56.48%	53.89%	73.68%	55.10%	73.95%
Linear SVM	50.11%	42.36%	78.89%	31.93%	55.09%	63.65%
RBF_SVM	61.94%	60.00%	1.67%	100%	5.41%	82.89%
RVM	64.73%	58.94%	30.00%	86.67%	39.57%	-
MLP-Net	61.94%	50.56%	79.44%	50.88%	61.57%	69.92%
FC-Net	57.20%	46.18%	69.44%	49.47%	54.91%	60.65%
ELM	63.01%	52.40%	49.44%	71.58%	50.82%	-
KNN	47.31%	42.10%	96.11%	16.49%	58.55%	66.45%
Bagged Trees	60.22%	44.09%	11.11%	91.23%	17.45%	57.53%
Boosted Trees	60.00%	46.42%	25.56%	81.75%	32.85%	57.58%
Boosted Stumps	60.00%	44.84%	22.22%	83.86%	29.48%	58.62%
Random Forest	61.29%	0%	0%	100%	-	58.04%
Rotation Forest	61.72%	56.00%	4.44%	97.89%	10.05%	-
BrainNetCNN	71.62%	66.56%	54.44%	82.44%	58.50%	74.74%
GAN	73.96%	72.80%	55.02%	85.96%	61.22%	76.34%
GCN	75.50%	71.66%	61.12%	84.58%	65.48%	78.80%

**Table 2:** Performance comparison of models on ABIDE-II dataset for multinomial (**a**) and binary (**b**) classification (Red color indicates best performance, while blue color denotes second best performance).

			(**a**)			
**Model**	**Accuracy**	**Precision**	**Recall**	**Specificity**	**F1 Score**	**AUC**
GNB	66.13%	47.83%	41.90%	73.89%	44.54%	68.13%
LDS	64.52%	46.19%	31.33%	81.67%	37.25%	65.89%
QDA	46.45%	21.91%	27.05%	55.56%	24.18%	53.09%
SLR	71.29%	43.23%	33.90%	85.00%	37.14%	77.96%
RLR	70.97%	49.86%	40.29%	80.56%	43.68%	77.96%
Linear SVM	71.29%	47.21%	40.29%	81.11%	43.07%	75.13%
RBF_SVM	63.23%	38.12%	10.48%	96.67%	16.32%	72.00%
RVM	69.68%	72.68%	33.24%	88.33%	45.16%	-
MLP-Net	65.16%	34.72%	39.81%	71.11%	36.89%	74.56%
FC-Net	56.13%	17.78%	24.76%	67.78%	24.50%	63.67%
ELM	58.71%	27.45%	33.05%	66.11%	29.98%	-
KNN	59.68%	37.00%	4.76%	97.22%	8.13%	58.99%
Bagged Trees	55.81%	18.52%	11.90%	82.22%	14.27%	54.14%
Boosted Trees	57.42%	22.17%	14.76%	81.67%	17.49%	59.26%
Boosted Stumps	60.03%	27.18%	19.05%	81.67%	22.32%	59.44%
Random Forest	60.32%	34.29%	6.19%	96.67%	10.23%	61.84%
Rotation Forest	59.03%	22.90%	12.38%	87.22%	15.89%	-
BrainNetCNN	70.36%	39.28%	28.20%	90.02%	33.12%	70.5%
GAN	73.56%	34.7%	32.88%	88.34%	33.60%	68.26%
GCN	72.28%	38.78%	32.02%	88.90%	34.96%	72.68%
			(**b**)			
**Model**	**Accuracy**	**Precision**	**Recall**	**Specificity**	**F1 Score**	**AUC**
GNB	69.35%	63.65%	63.08%	73.89%	63.33%	72.29%
LDS	68.06%	65.99%	49.23%	81.67%	56.29%	71.60%
QDA	56.45%	48.20%	57.69%	55.56%	52.48%	56.62%
SLR	73.55%	73.62%	57.69%	85.00%	64.65%	81.11%
RLR	74.52%	71.05%	66.15%	80.56%	68.50%	80.34%
Linear SVM	74.52%	71.55%	65.38%	81.11%	68.19%	81.54%
RBF_SVM	63.55%	79.10%	17.69%	96.67%	28.74%	80.21%
RVM	71.94%	75.48%	49.23%	88.33%	59.41%	-
MLP-Net	70.32%	63.98%	69.23%	71.11%	66.04%	77.28%
FC-Net	60.00%	53.84%	49.23%	67.78%	44.79%	65.19%
ELM	66.13%	58.51%	66.15%	66.11%	62.04%	-
KNN	59.68%	74.00%	7.69%	97.22%	13.51%	58.53%
Bagged Trees	58.71%	51.06%	26.15%	82.22%	34.09%	57.79%
Boosted Trees	59.03%	51.78%	27.69%	81.67%	35.62%	59.17%
Boosted Stumps	61.29%	57.26%	33.08%	81.67%	41.84%	53.95%
Random Forest	61.29%	79.43%	12.31%	96.67%	20.88%	65.38%
Rotation Forest	60.97%	56.69%	24.61%	87.22%	33.95%	-
BrainNetCNN	73.56%	78.64%	50.58%	90.02%	61.54%	75.84%
GAN	77.40%	79.62%	62.30%	88.34%	69.62%	75.90%
GCN	75.16%	78.44%	56.12%	88.90%	65.06%	74.12%

**Table 3:** Performance comparison of models on PTSD dataset for multinomial (**a**) and binary (**b**) classification (Red color indicates best performance, while blue color denotes second best performance).

			(**a**)			
**Model**	**Accuracy**	**Precision**	**Recall**	**Specificity**	**F1 Score**	**AUC**
GNB	82.22%	81.89%	78.75%	80%	80.29%	92.75%
LDS	50.00%	49.27%	57.50%	43.33%	52.96%	68.35%
QDA	47.78%	44.44%	40.00%	53.33%	41.67%	58.79%
SLR	88.89%	90.83%	81.25%	93.33%	85.57%	98.81%
RLR	95.56%	94.53%	93.33%	93.33%	95.29%	99.53%
Linear SVM	96.64%	97.78%	96.25%	96.67%	96.95%	99.53%
RBF_SVM	68.89%	59.45%	57.50%	63.33%	56.66%	97.94%
RVM	68.89%	75.00%	67.50%	56.67%	70.31%	-
MLP-Net	92.22%	91.75%	96.25%	86.67%	93.78%	98.56%
FC-Net	68.89%	52.61%	48.75%	86.67%	48.10%	94.01%
ELM	36.67%	44.09%	45.00%	23.33%	43.97%	-
KNN	48.89%	23.52%	48.75%	16.67%	31.71%	73.63%
Bagged Trees	83.33%	90.02%	77.50%	86.67%	83.08%	91.72%
Boosted Trees	70.00%	80.46%	68.75%	56.67%	74.00%	85.88%
Boosted Stumps	60.00%	66.10%	65.00%	30.00%	64.34%	76.87%
Random Forest	81.11%	85.76%	77.5%	73.33%	81.12%	97.51%
Rotation Forest	83.33%	87.71%	78.75%	80.00%	82.49%	-
BrainNetCNN	97.76%	98.88%	97.50%	96.67%	98.08%	98.44%
GAN	96.64%	95.76%	98.76%	93.33%	97.20%	98.98%
GCN	95.56%	95.76%	95%	93.33%	95.32%	96.96%
(**b**)						
**Model**	**Accuracy**	**Precision**	**Recall**	**Specificity**	**F1 Score**	**AUC**
GNB	90.00%	90.51%	95.00%	80.00%	92.67%	94.72%
LDS	68.89%	74.49%	81.67%	43.33%	77.81%	64.72%
QDA	64.44%	74.62%	70.00%	53.33%	71.94%	61.67%
SLR	96.67%	96.79%	98.33%	93.33%	97.53%	98.61%
RLR	97.76%	96.92%	100.00%	93.33%	98.40%	99.44%
Linear SVM	97.76%	98.46%	98.33%	96.67%	98.33%	99.44%
RBF_SVM	87.78%	84.57%	100.00%	63.33%	91.62%	98.33%
RVM	80.00%	80.95%	91.67%	56.67%	85.90%	-
MLP-Net	94.44%	94.33%	98.33%	86.67%	96.11%	98.33%
FC-Net	75.56%	94.18%	70.00%	86.67%	76.09%	93.06%
ELM	54.44%	64.94%	70.00%	23.33%	66.78%	-
KNN	70.00%	69.90%	96.67%	16.67%	81.06%	83.06%
Bagged Trees	90.00%	93.85%	91.67%	86.67%	92.51%	92.78%
Boosted Trees	80.00%	81.42%	91.67%	56.67%	85.92%	86.11%
Boosted Stumps	66.67%	68.75%	91.67%	16.67%	78.57%	75.00%
Random Forest	90.00%	88.22%	98.33%	73.33%	92.92%	99.44%
Rotation Forest	90.00%	90.58%	95.00%	80.00%	92.59%	-
BrainNetCNN	97.76%	98.46%	98.33%	96.67%	98.34%	98.60%
GAN	97.76%	96.92%	100.00%	93.33%	98.40%	99.16%
GCN	97.76%	96.92%	100.00%	93.33%	98.40%	96.38%

**Table 4:** Performance comparison of models on ADNI dataset for multinomial (**a**) and binary (**b**) classification (Red color indicates best performance, while blue color denotes second best performance).

			(**a**)			
**Model**	**Accuracy**	**Precision**	**Recall**	**Specificity**	**F1 Score**	**AUC**
GNB	37.14%	38.00%	27.78%	55.00%	31.82%	55.85%
LDS	30.00%	40.78%	32.78%	25.00%	36.15%	57.00%
QDA	22.86%	25.52%	28.89%	10.00%	26.49%	49.22%
SLR	32.86%	23.22%	21.67%	65.00%	21.88%	58.34%
RLR	32.86%	28.78%	28.89%	45.00%	27.86%	62.16%
Linear SVM	35.71%	29.89%	29.44%	50.00%	29.45%	57.95%
RBF_SVM	30.00%	24.02%	17.78%	55.00%	19.26%	63.80%
RVM	37.14%	40.33%	32.78%	50.00%	35.95%	-
MLP-Net	37.14%	32.78%	36.67%	35.00%	34.49%	59.52%
FC-Net	35.71%	23.94%	31.11%	50.00%	26.84%	66.11%
ELM	17.14%	21.44%	21.67%	5.00%	20.06%	-
KNN	24.29%	23.00%	30.56%	5.00%	26.11%	50.59%
Bagged Trees	24.29%	10.89%	13.33%	50.00%	19.95%	54.45%
Boosted Trees	25.71%	29.75%	26.67%	25.00%	34.00%	52.33%
Boosted Stumps	30.00%	36.83%	39.44%	10.00%	36.33%	52.33%
Random Forest	37.14%	41.22%	29.44%	55.00%	34.15%	54.57%
Rotation Forest	30.00%	42.00%	25.00%	40.00%	31.16%	-
BrainNetCNN	38.02%	21.50%	23.34%	50.00%	21.66%	58.86%
GAN	52.84%	42.42%	41.66%	80.00%	41.20%	66.42%
GCN	44.28%	37.56%	29.46%	55.00%	31.82%	62.46%
			(**b**)			
**Model**	**Accuracy**	**Precision**	**Recall**	**Specificity**	**F1 Score**	**AUC**
GNB	65.71%	79.72%	70.00%	55.00%	74.50%	59.00%
LDS	58.57%	70.73%	72.00%	25.00%	71.33%	74.50%
QDA	54.29%	66.57%	72.00%	10.00%	68.99%	41.00%
SLR	74.29%	84.67%	78.00%	65.00%	81.16%	85.00%
RLR	75.71%	80.51%	88.00%	45.00%	83.86%	87.00%
Linear SVM	71.43%	80.41%	80.00%	50.00%	79.94%	81.00%
RBF_SVM	65.71%	79.72%	70.00%	55.00%	74.50%	70.50%
RVM	70.00%	79.78%	78.00%	50.00%	78.84%	-
MLP-Net	78.57%	79.34%	96.00%	35.00%	86.60%	88.00%
FC-Net	74.29%	81.14%	84.00%	50.00%	82.22%	80.00%
ELM	50.00%	63.84%	68.00%	5.00%	65.58%	-
KNN	55.71%	66.73%	76.00%	5.00%	70.97%	59.50%
Bagged Trees	60.00%	77.17%	64.00%	50.00%	69.60%	70.00%
Boosted Trees	65.71%	73.41%	82.00%	25.00%	76.20%	69.00%
Boosted Stumps	65.71%	71.00%	88.00%	10.00%	78.36%	53.50%
Random Forest	65.71%	79.43%	70.00%	55.00%	73.88%	70.75%
Rotation Forest	60.00%	74.11%	68.00%	40.00%	70.81%	-
BrainNetCNN	82.86%	82.88%	96.00%	50.00%	88.80%	82.00%
GAN	88.56%	92.66%	92.00%	80.00%	91.96%	84.00%
GCN	80.00%	83.47%	90.00%	55.00%	86.34%	84.18%

**Table 5:** The *p*-values of the Bernoulli test for all the models. Significance was defined at α=0.05.

Model	Dataset			
	ADHD	ABIDE-II	PTSD	ADNI
GNB	0.005	0.001	4.84 $\times \;10^{-4}$	0.042
LDA	0.175	0.003	0.079	0.168
QDA	0.302	0.155	0.079	0.282
SLR	0.008	6.95 $\times \;10^{-5}$	8.12 $\times \;10^{-5}$	0.006
RLR	0.001	6.95 $\times \;10^{-5}$	1.10 $\times \;10^{-5}$	0.006
LinearSVM	0.459	6.95 $\times \;10^{-5}$	1.10 $\times \;10^{-5}$	0.017
RBF-SVM	0.009	0.021	4.84 $\times \;10^{-4}$	0.042
RVM	0.003	1.88 $\times \;10^{-4}$	0.009	0.017
MLP-Net	0.008	4.80 $\times \;10^{-4}$	8.12 $\times \;10^{-5}$	0.002
FC-Net	0.089	0.064	0.009	0.006
ELM	0.005	0.005	0.319	0.424
kNN	0.698	0.064	0.030	0.282
Bagged Tree	0.024	0.102	4.84 $\times \;10^{-4}$	0.168
Boosted Tree	0.024	0.064	0.009	0.042
Boosted Stump	0.024	0.038	0.003	0.042
Random Forest	0.015	0.038	4.84 $\times \;10^{-4}$	0.042
Rotation Forest	0.015	0.038	4.84 $\times \;10^{-4}$	0.168
BrainNetCNN	1.06 $\times \;10^{-5}$	6.95 $\times \;10^{-5}$	1.10 $\times \;10^{-5}$	5.35 $\times \;10^{-5}$
GCN	5.48 $\times \;10^{-7}$	2.41 $\times \;10^{-5}$	1.10 $\times \;10^{-5}$	5.35 $\times \;10^{-5}$
GAN	1.53 $\times \;10^{-6}$	7.87 $\times \;10^{-6}$	1.10 $\times \;10^{-5}$	2.66 $\times \;10^{-5}$

**Table 6:** The *p*-value of the Wilcoxon rank-sum test for the comparisons of GAN with the other models (**a**) and GCN with the other models (**b**) on all the datasets. Significance was defined at α<0.05.

		(**a**)		
**Model**	**Dataset**			
	**ADHD**	**ABIDE-II**	**PTSD**	**ADNI**
GNB	0.004	0.004	0.04	0.004
LDA	0.004	0.004	0.004	0.004
QDA	0.004	0.004	0.004	0.004
SLR	0.004	0.032	0.579	0.020
RLR	0.004	0.032	0.738	0.004
LinearSVM	0.004	0.059	0.738	0.004
RBF-SVM	0.004	0.004	0.004	0.004
RVM	0.004	0.004	0.004	0.004
MLP-Net	0.004	0.044	0.341	0.171
FC-Net	0.004	0.004	0.004	0.004
ELM	0.004	0.004	0.004	0.004
kNN	0.004	0.004	0.004	0.004
Bagged Tree	0.004	0.004	0.087	0.004
Boosted Tree	0.004	0.004	0.004	0.004
Boosted Stump	0.004	0.004	0.004	0.004
Random Forest	0.004	0.004	0.012	0.004
Rotation Forest	0.004	0.004	0.04	0.004
BrainNetCNN	0.206	0.020	0.738	0.198
GCN	0.794	0.187	0.738	0.059
		(**b**)		
**Model**	**Dataset**			
	**ADHD**	**ABIDE-II**	**PTSD**	**ADNI**
GNB	0.004	0.004	0.04	0.008
LDA	0.004	0.004	0.004	0.008
QDA	0.004	0.004	0.004	0.004
SLR	0.004	0.14	0.579	0.159
RLR	0.004	0.38	0.738	0.048
LinearSVM	0.004	0.556	0.738	0.044
RBF-SVM	0.004	0.004	0.004	0.008
RVM	0.004	0.016	0.004	0.044
MLP-Net	0.004	0.194	0.341	0.567
FC-Net	0.004	0.004	0.004	0.044
ELM	0.004	0.008	0.004	0.004
kNN	0.004	0.004	0.004	0.004
Bagged Tree	0.004	0.004	0.087	0.008
Boosted Tree	0.004	0.004	0.004	0.016
Boosted Stump	0.004	0.004	0.004	0.012
Random Forest	0.004	0.004	0.012	0.016
Rotation Forest	0.004	0.004	0.04	0.004
BrainNetCNN	0.095	0.258	0.738	0.825
GAN	0.270	0.877	0.738	1

**Table 7:** The comparison of computational time (in seconds) required to train each model.

Model	Dataset			
	ADHD	ABIDE-II	PTSD	ADNI
GNB	0.12	0.11	0.01	0.01
LDA	1.65	1.49	0.22	0.34
QDA	6.28	3.09	0.07	0.1
SLR	44.18	29.14	2.18	5.35
RLR	21.29	14.88	1.41	3.54
LinearSVM	67.83	4.22	3.08	0.48
RBF-SVM	5.13	1.61	0.05	0.3
RVM	242.32	92.47	35.31	23.24
MLP-Net	24.09	19.55	10.77	8.19
FC-Net	18.86	17.26	10.17	9.42
ELM	2.02	0.08	0.17	0.17
kNN	0.409	0.23	0.01	0.01
Bagged Tree	52.74	35.92	1.32	2.79
Boosted Tree	5.06	4.40	0.388	0.63
Boosted Stump	4.02	3.99	0.41	0.55
Random Forest	5.20	3.23	0.29	0.30
Rotation Forest	304.66	201.79	19.89	34.10
BrainNetCNN	114.42	133.79	38.74	132.46
GCN	144.6	110.57	34.13	90.76
GAN	194.98	236.23	83.29	260.87

## Data Availability

The data from ADHD (https://fcon_1000.projects.nitrc.org/indi/adhd200/, accessed on 19 March 2024), Autism (https://fcon_1000.projects.nitrc.org/indi/abide/, accessed on 19 March 2024) and Alzheimer’s (https://adni.loni.usc.edu/, accessed on 19 March 2024) is available publicly and we have used those public datasets. The PTSD data were acquired in-house and were funded by the US Department of Defense. Contractual obligations do not allow us to publicly share the raw data; however, we are happy to share processed data with individual investigators upon request.
